# Favorable Effects of GLP-1 Receptor Agonist against Pancreatic β-Cell Glucose Toxicity and the Development of Arteriosclerosis: “The Earlier, the Better” in Therapy with Incretin-Based Medicine

**DOI:** 10.3390/ijms22157917

**Published:** 2021-07-24

**Authors:** Hideaki Kaneto, Tomohiko Kimura, Masashi Shimoda, Atsushi Obata, Junpei Sanada, Yoshiro Fushimi, Shuhei Nakanishi, Tomoatsu Mune, Kohei Kaku

**Affiliations:** 1 Department of Diabetes, Endocrinology and Metabolism, Kawasaki Medical School, Kurashiki 701-0192, Japan; tomohiko@med.kawasaki-m.ac.jp (T.K.); masashi-s@med.kawasaki-m.ac.jp (M.S.); obata-tky@med.kawasaki-m.ac.jp (A.O.); gengorou@med.kawasaki-m.ac.jp (J.S.); fussy.k0113@med.kawasaki-m.ac.jp (Y.F.); nshuhei@med.kawasaki-m.ac.jp (S.N.); mune@med.kawasaki-m.ac.jp (T.M.); 2 General Medical Center, Kawasaki Medical School, Kurashiki 701-0192, Japan; kka@med.kawasaki-m.ac.jp

**Keywords:** pancreatic β-cells, glucose toxicity, arteriosclerosis, GLP-1 receptor agonist, incretin-based medicine

## Abstract

Fundamental pancreatic β-cell function is to produce and secrete insulin in response to blood glucose levels. However, when β-cells are chronically exposed to hyperglycemia in type 2 diabetes mellitus (T2DM), insulin biosynthesis and secretion are decreased together with reduced expression of insulin transcription factors. Glucagon-like peptide-1 (GLP-1) plays a crucial role in pancreatic β-cells; GLP-1 binds to the GLP-1 receptor (GLP-1R) in the β-cell membrane and thereby enhances insulin secretion, suppresses apoptotic cell death and increase proliferation of β-cells. However, GLP-1R expression in β-cells is reduced under diabetic conditions and thus the GLP-1R activator (GLP-1RA) shows more favorable effects on β-cells at an early stage of T2DM compared to an advanced stage. On the other hand, it has been drawing much attention to the idea that GLP-1 signaling is important in arterial cells; GLP-1 increases nitric oxide, which leads to facilitation of vascular relaxation and suppression of arteriosclerosis. However, GLP-1R expression in arterial cells is also reduced under diabetic conditions and thus GLP-1RA shows more protective effects on arteriosclerosis at an early stage of T2DM. Furthermore, it has been reported recently that administration of GLP-1RA leads to the reduction of cardiovascular events in various large-scale clinical trials. Therefore, we think that it would be better to start GLP-1RA at an early stage of T2DM for the prevention of arteriosclerosis and protection of β-cells against glucose toxicity in routine medical care.

## 1. Introduction

The number of patients with type 2 diabetes mellitus (T2DM) has been increasing all over the world and T2DM is recognized as one of the most prevalent and serious metabolic diseases. In addition, economic and healthcare burden due to T2DM is a matter of concern at present. Therefore, it is very important to clarify the molecular mechanism for the pathophysiology of T2DM. Two main characteristics of T2DM are the pancreatic β-cell dysfunction and insulin resistance in various insulin target tissues such as the liver, skeletal muscle and adipose tissues. Normal β-cells can compensate for insulin resistance by increasing insulin secretion or β-cell mass, but insufficient compensation leads to the onset of T2DM. After then, once hyperglycemia becomes apparent, the β-cell function gradually deteriorates and insulin resistance aggravates.

**Figure 1:**
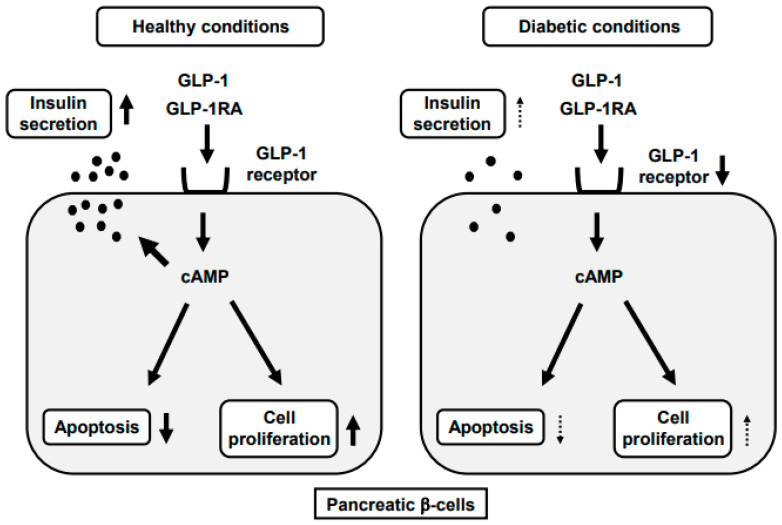
Reduction of GLP-1RA effects on pancreatic β-cells under diabetic conditions. GLP-1 binds to its receptor in pancreatic β-cells, which leads to the enhancement of insulin secretion, reduction of apoptotic cell death and increase of β-cell proliferation. After chronic exposure to hyperglycemia, however, GLP-1 receptor expression in β-cells is reduced, which weakens the protective effects of GLP-1 and GLP-1RA against β-cells glucose toxicity.

It is well known that incretins, glucagon-like peptide-1 (GLP-1) and glucose-dependent insulinotropic polypeptide (GIP) have pleiotropic effects on a variety of tissues including pancreatic β-cells, artery, heart, liver, neuron and adipose tissues. While there are various GLP-1 target tissues, GLP-1 plays a crucial role in pancreatic β-cells; GLP-1 binds to the GLP-1 receptor (GLP-1R) in the β-cell membrane and thereby enhances insulin secretion, suppresses apoptotic cell death and increases the proliferation of β-cells. However, GLP-1R expression is reduced under diabetic conditions (Figure 1). The glucagon-like peptide-1 receptor agonist (GLP-1RA) and dipeptidyl peptidase-IV (DPP-IV) inhibitor are very often used in subjects with type 2 diabetes mellitus (T2DM). The DPP-IV inhibitor suppresses activity of DPP-IV, which is a splitting enzyme of incretin and increases serum levels of GLP-1 and GIP. Both incretins stimulate insulin secretion in a glucose-dependent manner and GLP-1 suppresses glucagon secretion, leading to the amelioration of glycemic control. Furthermore, the GLP-1RA and DPP-IV inhibitor do not usually bring about hypoglycemia and/or weight gain. GLP-1RA markedly increases circulating GLP-1 levels and functions at very high concentrations. Thereby, GLP-1RA has more potent glucose-lowering effects compared to the DPP-IV inhibitor. In addition, GLP-1RA reduces body weight by increasing central satiety and delaying gastric emptying. On the other hand, it has been drawing much attention to the idea that GLP-1 signaling is important in arteries as well. Indeed, it has been reported recently that administration of GLP-1RA leads to the reduction of cardiovascular events.

## 2. Incretin and Pancreatic β-Cells

T2DM is characterized by pancreatic β-cell dysfunction and insulin resistance. It has been shown that chronic hyperglycemia leads to the decrease of insulin biosynthesis and secretion due to β-cell glucose toxicity [1,2,3,4,5,6,7,8,9,10,11,12] and that the reduction of insulin mRNA expression is accompanied by decreased nuclear expression of insulin transcription factors such as MafA and PDX-1 [13,14,15,16,17,18,19,20,21]. Such phenomena are known as β-cell glucose toxicity. It has been shown, however, that β-cell function is recovered by the treatment with various antidiabetes medicine at an early stage of T2DM to some extent [22,23,24,25,26,27].

In response to the ingestion of food, GLP-1 and GIP are secreted from the gastrointestinal tract and stimulate insulin secretion from pancreatic β-cells. Both incretin hormones bind to each receptor in the β-cell membrane, which leads to enhancing insulin secretion, reducing β-cell apoptosis and promoting β-cell proliferation. Such an action of incretin hormones, however, is significantly reduced under diabetic conditions in humans and rodents such as mice and rats. It has been reported that expression levels of incretin receptors are reduced under diabetic conditions, which is probably involved in the impaired incretin effects and the development of β-cell failure found in T2DM [28,29,30]. For example, such downregulation of incretin receptor expression has been shown in obese type 2 diabetic mice (at 16 weeks of age, non-fasting blood glucose levels were about 500 mg/dL) and 90% partial pancreatectomized rats (4 weeks after the operation, fasting blood glucose levels were about 200 mg/dL). The precise mechanism for reduction of GLP-1 and GIP receptor levels under diabetic conditions remained unraveled. It has been shown recently, however, that the reduction of the transcription factor 7-like 2 (TCF7L2) expression level, which is a transcription factor and plays a crucial role in the maintenance of β-cell function, is involved in the downregulation of incretin receptor expression in β-cells [31,32,33]. It has been reported that TCF7L2 is involved in insulin biosynthesis, secretion and preservation of β-cell mass through the AKT and mTOR pathway [31,32,33,34,35,36,37]. Indeed, it is known that inactivation of TCF7L2 leads to the impairment of insulin secretion and glucose tolerance. Since TCF7L2 is a downstream factor transcription of the β-catenin signaling pathway, TCF7L2 is physiologically regulated by β-catenin. In addition, TCF7L2 is regulated by its genetic variation. Indeed, it is known that common genetic variations of TCF7L2 are associated with T2DM and that the subjects with the high-risk allele of TCF7L2 show impaired insulin secretion [38,39,40,41,42].

## 3. GLP-1-Stimulated Insulin Secretion

Insulin secretion is regulated by various intracellular signals in β-cells. Among them, cyclic adenosine monophosphate (cAMP) is particularly important for amplifying insulin secretion. While GLP-1RA and DPP-IV inhibitors have been often used in clinical practice, such incretin-based drugs function through the cAMP signaling. It is thought that cAMP potentiates insulin secretion through protein kinase A (PKA) phosphorylation of factors, which are associated with insulin secretory process. However, it has been proposed that there is another pathway for cAMP-induced insulin secretion; it was shown that cAMP had another target named Epac (also called as cAMP-GEF) in β-cells [43,44,45,46]. It is known that Epac signaling regulates cAMP-induced insulin granule exocytosis through the enlargement of the size of a readily released pool.

In addition, it was reported recently that a physiologically low concentration of GLP-1 activated protein kinase C (PKC) without a significant increase of intracellular cAMP, which also led to the enhancement of insulin secretion [47,48]. Thereby, it is likely that GLP-1 stimulates insulin secretion in a PKC-dependent or PKA-dependent manner, depending on its concentration. They showed that GLP-1 increased intracellular diacylglycerol and Ca^2+^ and activated PKC, leading to membrane depolarization and subsequent stimulation of insulin secretion. They also showed that the depolarizing effect of GLP-1 on electrical activity was mimicked by a PKC activator without activation of the PKA pathway. These new findings clearly indicate that circulating physiological concentration of GLP-1 directly stimulates insulin secretion from pancreatic β-cells.

## 4. GLP-1RA and Pancreatic β-Cells

Incretin-based medicine such as the GLP-1RA and DPP-IV inhibitor ameliorate glycemic control and mitigate the progression of β-cell dysfunction in human subjects and animal models. It has been reported that GLP-1RA preserves pancreatic β-cells in various types of T2DM rodents [49,50,51,52,53,54,55]. For example, it was shown that when T2DM db/db mice at 10 weeks old were treated with GLP-1RA (liraglutide) for 2 weeks, metabolic variables and insulin sensitivity were improved. GLP-1RA also increased glucose-stimulated insulin secretion (GSIS) and islet insulin content and reduced triglyceride content in islets. Furthermore, expression levels of various genes related to proapoptosis, ER stress and lipid synthesis were downregulated whereas those related to antiapoptosis and antioxidative stress were upregulated. GLP-1RA treatment for 2 days slightly improved metabolic variables in db/db mice, but GSIS, insulin and triglyceride content were not affected. Such treatment increased gene expression related to cell differentiation, proliferation and antiapoptosis and suppressed gene expression involved in proapoptosis, although there was no effect on oxidative stress- or ER stress-related factors [49]. Taken together, GLP-1RA increases β-cell mass not only by directly regulating cell kinetics, but also by suppressing oxidative and ER stress, secondary to the amelioration of glucolipotoxicity.

Protective effects of GLP-1RA on β-cells were reported in another type of diabetic mice [52,53,54,55]. For example, it was shown that GLP-1RA improved pancreatic β-cell mass and function in alloxan-induced diabetic mice [52]. They examined the effects of GLP-1RA on β-cell fate and function by using an inducible Cre/loxP system. In the results, chronic GLP-1RA treatment for 30 days improved glucose tolerance and insulin response to oral glucose load. Additionally, GLP-1RA treatment doubled β-cell mass compared to the vehicle group by increasing the β-cell proliferation rate and reducing apoptotic cell death. Interestingly, however, there was no or little contribution of neogenesis to such an increase in β-cell mass based on the data obtained with the Cre/loxP system. In addition, GLP-1RA reduced oxidative stress in pancreatic islets. Furthermore, the beneficial effects of GLP-1RA in these mice were maintained 2 weeks after drug withdrawal [52]. In another study, it was shown in more detail how GLP-1RA preserved β-cell mass [54]. They showed that GLP-1RA protected mouse pancreatic β-cell line βTC6 cells from serum withdrawal-induced apoptosis through the inactivation of caspase-3. They also showed that PI3-kinase-dependent AKT phosphorylation, inactivation of the proapoptotic protein BAD and inhibition of the FoxO1 transcription factor were involved in antiapoptotic action of GLP-1RA [54].

It has been reported that GLP-1RA shows more favorable effects at an early stage of T2DM compared to an advanced stage [50,51]. T2DM db/db mice were treated with GLP-1RA (liraglutide) and/or pioglitazone for 2 weeks at an early (7 weeks old) and advanced stage (16 weeks old). At an early stage, insulin biosynthesis and secretion were markedly increased by such a treatment, which was not observed at an advanced stage. Expression levels of various β-cell-related factors such as MafA and PDX-1 were upregulated by such a treatment only at an early stage. It is likely that the recovery of MafA expression after such treatment is particularly important for the recovery of the β-cell function and amelioration of glycemic control, because MafA regulates not only insulin but also various factors related to GSIS. The increased expression of GLUT2 and glucokinase could also explain the augmentation of GSIS observed at an early stage. In addition, the expression level of GLP-1R was reduced at an advanced stage, which we think explains the reason why GLP-1RA showed more favorable effects at an early stage. Furthermore, β-cell mass and proliferation were increased by the treatment only at an early stage. [51]. Similarly, it was shown that DPP-IV inhibitor together with the SGLT2 inhibitor exerted more favorable effects on the β-cell function and mass at an early stage of T2DM compared to an advanced stage [56]. It is well known that GLP-1 binds to its receptor in the β-cell membrane and activates adenylate cyclase and the cAMP/PKA signaling pathway [43,44,45,46]. In addition, a low concentration of GLP-1 activates PKC without a significant increase of intracellular cAMP, which also leads to the enhancement of insulin secretion [47,48]. It is known that the activation of such kinases is involved in the reduction of β-cell apoptosis, proliferation of β-cells and maintenance of β-cell function. In this study, expression of the GLP-1R level was downregulated at an advanced stage compared with an early stage. Therefore, we think that the downregulation of signal pathways such as PKA or PKC is, at least in part, involved in the ineffectiveness of GLP-1RA on the β-cell function at an advanced stage. Taken together, the usage of incretin-based medicine at an early stage of T2DM would be useful and promising for the preservation of the β-cell function and mass.

On the other hands, it is well known that chronic exposure to a large amount of ligand leads to the downregulation of its receptor. In addition, it is known that the serum GLP-1 level becomes very high after the usage of GLP-1RA, which is a ligand of GLP-1R. It remained unknown, however, whether the long-time usage of GLP-1RA downregulates its receptor. It was reported that GLP-1R expression was reduced after long-term exposure to GLP-1RA (dulaglutide) in non-diabetic and diabetic mice. Obese type 2 diabetic db/db mice and non-diabetic db/m mice were treated with GLP-1RA or the control vehicle for 17 weeks (from 7 to 24 weeks of age). Various metabolic parameters such as GSIS, insulin and triglyceride content in islets and β-cell-related gene expression were evaluated after the intervention. In db/m mice, GLP-1R expression in β-cells was not decreased even after long-term administration of GLP-1RA. In db/db mice, GLP-1R expression at 24 weeks of age was significantly lower compared to that at 7 weeks probably due to glucose toxicity [57]. Furthermore, GLP-1R expression in 24-week-old db/db mice treated with GLP-1RA was higher, rather than downregulated, compared to 24-week-old untreated diabetic mice, which was probably due to the amelioration of glycemic control. Food intake and blood glucose levels in db/db mice treated with GLP-1RA were lower until 24 weeks of age compared to untreated db/db mice. Expression levels of various β-cell-related genes, insulin biosynthesis and secretion were enhanced after GLP-1RA treatment in db/db mice. In contrast, oxidative and endoplasmic reticulum stress, inflammation, fibrosis and apoptosis were suppressed after GLP-1RA treatment [57]. Taken together, GLP-1RA shows favorable effects on glycemic control and protective effects on pancreatic β-cells for a long period without reducing the GLP-1R expression level.

GLP-1R is one of the G protein-coupled receptors (GPCRs). GLP-1 binds to its receptor on the cell membrane and the complex of GLP-1 and its receptor GPCR is internalized into cells. In general, it is thought that the internalized receptor preserves its expression level compared to a non-internalized receptor. Consequently, we think that such characteristics of GLP-1R could explain the reason why GLP-1R expression was not decreased even after long-term administration of GLP-1RA. In addition, some drug therapy has been developed by utilizing the phenomena that chronic exposure to a large amount of ligand downregulates its receptor expression. For example, in the treatment for endometriosis, the gonadotropin releasing hormone (GnRH) agonist suppresses the production of the downstream hormone through downregulation of its receptor by continuous administration of a large amount of ligand [58,59,60,61].

## 5. GLP-1RA and Arteriosclerosis

### 5.1. Incretin Signaling and Arterial Cells

**Figure 2:**
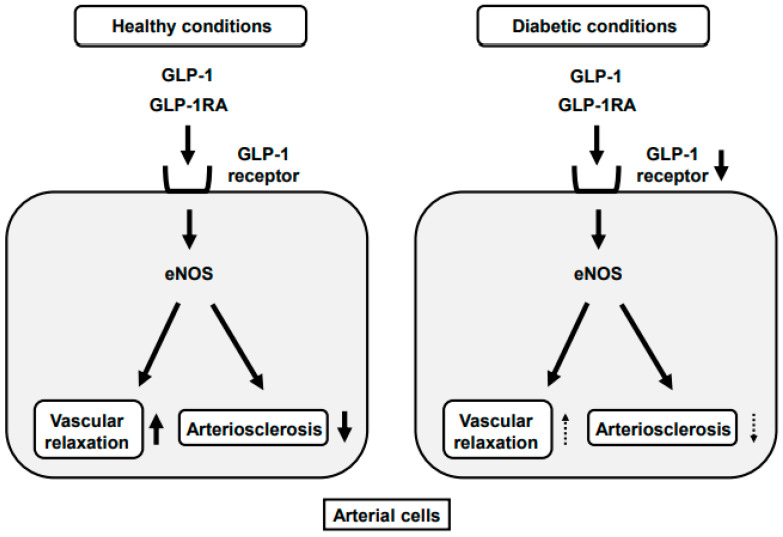
Reduction of GLP-1RA effects on arterial cells under diabetic conditions. GLP-1 binds to its receptor in arterial cells, which leads to the enhancement of vascular relaxation and the prevention of arteriosclerosis. After chronic exposure to hyperglycemia, however, GLP-1 receptor expression in arterial cells is reduced, which weakens the protective effects GLP-1 and GLP-1RA against arteriosclerosis.

GLP-1R expression is observed in endothelial and smooth muscle cells. In endothelial cells, incretin signaling improves the vascular relaxation response through eNOS expression and activity and retards the development of arteriosclerosis (Figure 2) [62,63,64]. Activation of GLP-1 signaling in arteries leads to the mitigation of inflammatory cytokines. In arterial cells, GLP-1 signaling improves the wall disorder induced by various factors including hyperglycemia and inflammatory cytokines. In vascular smooth muscle cells, GLP-1R stimulation prevents the development of arteriosclerosis. In addition, although GLP-1R is expressed in various cell types, it was not clearly elucidated how GLP-1RA can retard the progression of arteriosclerosis. However, recently the vasoprotective mechanism of GLP-1RA was clearly demonstrated at the cellular level by using global GLP-1R knockout mice, endothelial cell-specific GLP-1 knockout mice and myeloid cell-specific GLP-1R knockout mice. As the results, it was shown that GLP-1RA treatment normalized blood pressure, cardiac hypertrophy, vascular fibrosis, endothelial dysfunction, oxidative stress and vascular inflammation in an endothelial GLP-1R-dependent manner [65]. We think that these novel findings are strong evidence showing that endothelial GLP-1R expression is critical for GLP-1 to fully show their effects in arteries. Incretin-based therapy substantially ameliorates glycemic control without hypoglycemia and/or weight gain, which leads one to the prevention of diabetic macroangiopathy. In addition, GLP-1 has direct protective effects on vascular cells via GLP-1R. Therefore, it is likely that incretin-based therapy shows favorable effects on the development of arteriosclerosis through the reduction of blood glucose levels and their direct effects on arterial cells via GLP-1R.

### 5.2. Downregulation of GLP-1R Expression in Arterial Cells under Diabetic Conditions

GLP-1R expression in pancreatic β-cells is reduced under diabetic conditions and TCF7L2 is known to function as a transcription factor for GLP-1R at least in β-cells. Incretin signaling is known to prevent the development of arteriosclerosis by the relaxation response in endothelial cells via the GLP-1R. It was reported recently that GLP-1R and TCF7L2 expression levels in endothelial and smooth muscle cells were significantly lower in obese type 2 diabetic db/db mice compared to non-diabetic db/m mice [66]. Furthermore, reduction of the TCF7L2 level using siTCF7L2 resulted in the downregulation of GLP-1R expression in cultured vascular endothelial cells. In addition, when the TCF7L2 level was enhanced using the TCF7L2 expressing adenovirus, the GLP-1R expression level was substantially augmented [67]. In conclusion, the GLP-1R expression level was substantially reduced under diabetic conditions together with the decrease of the TCF7L2 level (Figure 2). Furthermore, it was shown that the TCF7L2 is a possible regulator of the GLP-1R expression in the artery as reported in β-cells.

### 5.3. Favorable Antiarteriosclerotic Effects of GLP-1RA

It was not known so far whether or not there was some difference in effectiveness of GLP-1RA between an early and an advanced stage of T2DM. Recently, however, to address such questions, either GLP-1RA (dulaglutide) or the vehicle was administered to streptozotocin-induced diabetic ApoE knockout mice from 10 to 18 weeks of age as an early stage and from 18 to 26 weeks as an advanced stage. In the results, in an early stage group, the arteriosclerotic lesion in the aortic arch and Mac-2 and CD68-positive areas in the aortic root were significantly smaller in the GLP-1RA group [68]. In the abdominal aorta, expression levels of various inflammation markers were lower in the GLP-1RA group. In an advanced stage group, there were no immunohistological differences in the aortic root and expression levels of various factors between the GLP-1RA and vehicle group [68]. Taken together, GLP-1RA shows more favorable antiarteriosclerotic effects at an early stage of T2DM compared to an advanced stage.

### 5.4. Protective Role of GLP-1RA against Cardiovascular Events in Subjects with T2DM

While cardiovascular events sometimes bring about serious and lethal situations, it has been shown recently that GLP-1RAs reduce cardiovascular events [69,70,71,72,73,74]. The LEADER trial showed the effects of a once-daily injection of GLP-1RA liraglutide on cardiovascular events. The primary composite cardiovascular outcome was observed in significantly fewer patients in the treatment group (hazard ratio (HR): 0.87). Additionally, fewer patients died from cardiovascular causes in the treatment group (HR: 0.78). The rate of the all-cause death was also lower in the treatment group (HR: 0.85) [69,70]. The REWIND trial showed the effects of a once-weekly injection of dulaglutide on cardiovascular events. HR in the primary composite cardiovascular outcome was 0.88 and that in the all-cause death was 0.90 [71,72]. The SUSTAIN-6 trial showed the effects of a once-weekly injection of semaglutide on cardiovascular events. The occurrence of the primary composite cardiovascular outcome was lower in the treatment group (HR: 0.74). HR in nonfatal myocardial infarction and nonfatal stroke was 0.74 and 0.61, respectively [73,74]. Taken together, a once-daily injection of liraglutide and once-weekly injection of dulaglutide and semaglutide are expected to prevent major adverse cardiovascular events. The above-mentioned three large-scale clinical trials strongly support the idea that GLP-1RAs show a protective role against cardiovascular events in subjects with T2DM. Therefore, in routine medical care we should willingly use GLP-1RA in subjects with T2DM especially in subjects with a large risk of cardiovascular events.

## 6. Conclusions

In this review article, we featured roles of GLP-1 signaling in pancreatic β-cells and arteries. In addition, we described the usability of GLP-1RA based on the molecular mechanism for β-cell glucose toxicity and for the development of arteriosclerosis.

Our current ideas about pancreatic β-cell glucose toxicity are as follows. First, chronic hyperglycemia leads to a decrease of insulin biosynthesis and/or secretion in the diabetic state, which is accompanied by decreased expression of insulin transcription factors. In routine medical care, it is very important to alleviate such β-cell glucose toxicity in order to prevent the aggravation of T2DM. Second, incretin signaling plays crucial roles in pancreatic β-cells and we believe that GLP-1RA is a promising medicine to protect β-cells against glucose toxicity. However, incretin sensitivity in β-cells is weakened under diabetic conditions, at least in part, due to downregulation of GLP-1R expression, which we think may be associated with the aggravation of β-cell glucose toxicity.

Our current ideas about the usability of GLP-1RA are as follows. First, as described above, GLP-1R expression in pancreatic β-cells is downregulated under diabetic conditions. The data also suggest that it would be better to use incretin-based medicine at an early stage of T2DM. Indeed, GLP-1RA showed more favorable effects on β-cells at an early stage in T2DM mice. Second, incretin signaling plays a crucial role against arteriosclerosis, but incretin sensitivity in arteries is also weakened, at least partially, due to downregulation of GLP-1R expression, which we think may facilitate the development of arteriosclerosis. Consequently, we think that it would be better to use incretin-based medicine at an early stage of T2DM for the prevention of arteriosclerosis. Indeed, GLP-1RA showed more favorable effects against the progression of arteriosclerosis at an early stage. Third, a series of large-scale clinical trials have shown that GLP-1RAs have favorable effects against the onset of cardiovascular events. Taken together, incretin signaling plays crucial roles in various kinds of cells such as pancreatic β-cells and arterial cells and GLP-1RAs are promising from the clinical points of view and basic research area.

## References

[1] Bensellam M., Laybutt D.R., Jonas J.C. (2012). The molecular mechanisms of pancreatic beta-cell glucotoxicity: Recent findings and future research directions. Mol. Cell. Endocrinol..

[2] Campos C. (2012). Chronic hyperglycemia and glucose toxicity: Pathology and clinical sequelae. Postgrad. Med..

[3] Weir G.C., Aguayo-Mazzucato C., Bonner-Weir S. (2013). beta-cell dedifferentiation in diabetes is important, but what is it?. Islets.

[4] Weir G.C., Bonner-Weir S. (2013). Islet beta cell mass in diabetes and how it relates to function, birth, and death. Ann. N. Y. Acad. Sci..

[5] Halban P.A., Polonsky K.S., Bowden D.W., Hawkins M.A., Ling C., Mather K.J., Powers A.C., Rhodes C.J., Sussel L., Weir G.C. (2014). β-Cell failure in type 2 diabetes: Postulated mechanisms and prospects for prevention and treatment. Diabetes Care.

[6] Alarcon C., Boland B.B., Uchizono Y., Moore P.C., Peterson B., Rajan S., Rhodes O.S., Noske A.B., Haataja L., Arvan P. (2016). Pancreatic β-cell adaptive plasticity in obesity increases insulin production but adversely affects secretory function. Diabetes.

[7] Boland B.B., Brown C., Boland M.L., Cann J., Sulikowski M., Hansen G., Grønlund R.V., King W., Rondinone C., Trevaskis J. (2019). Pancreatic beta-cell rest replenishes insulin secretory capacity and attenuates diabetes in an extreme model of obese type 2 diabetes. Diabetes.

[8] Lytrivi M., Castell A.L., Poitout V., Cnop M. (2020). Recent insights into mechanisms of beta-cell lipo- and glucolipotoxicity in Type 2 Diabetes. J. Mol. Biol..

[9] Hall E., Jonsson J., Ofori J.K., Volkov P., Perfilyev A., Dekker Nitert M., Eliasson L., Ling C., Bacos K. (2019). Glucolipotoxicity alters insulin secretion via epigenetic changes in human islets. Diabetes.

[10] Roma L.P., Jonas J.C. (2020). Nutrient Metabolism, Subcellular Redox State, and Oxidative Stress in Pancreatic Islets and beta-Cells. J. Mol. Biol..

[11] Benito-Vicente A., Jebari-Benslaiman S., Galicia-Garcia U., Larrea-Sebal A., Uribe K.B., Martin S. (2021). Molecular mechanisms of lipotoxicity-induced pancreatic beta-cell dysfunction. Int. Rev. Cell Mol. Biol..

[12] Hong J.H., Kim D.H., Lee M.K. (2021). Glucolipotoxicity and GLP-1 secretion. BMJ Open Diabetes Res. Care.

[13] Wang H., Brun T., Kataoka K., Sharma A.J., Wollheim C.B. (2007). MAFA controls genes implicated in insulin biosynthesis and secretion. Diabetologia.

[14] Matsuoka T., Kaneto H., Miyatsuka T., Yamamoto T., Yamamoto K., Kato K., Shimomura I., Stein R., Matsuhisa M. (2010). Regulation of MafA expression in pancreatic β-cells in db/db mice with diabetes. Diabetes.

[15] Hang Y., Yamamoto T., Benniger R.K., Brissova M., Guo M., Bush W., Piston D.W., Powers A.C., Magnuson M., Thurmond D.C. (2014). The MafA transcription factor becomes essential to islet β-cells soon after birth. Diabetes.

[16] Matsuoka T., Kaneto H., Kawashima S., Miyatsuka T., Tochino Y., Yoshikawa A., Imagawa A., Miyazaki J., Gannon M., Stein R. (2015). Preserving MafA expression in diabetic islet β-cells improves glycemic control in vivo. J. Biol. Chem..

[17] Kaneto H., Matsuoka T. (2015). Role of pancreatic transcription factors in maintenance of mature β-cell function. Int. J. Mol. Sci..

[18] Nishimura W., Takahashi S., Yasuda K. (2015). MafA is critical for maintenance of the mature beta cell phenotype in mice. Diabetologia.

[19] Yamamoto Y., Miyatsuka T., Sasaki S., Miyashita K., Kubo N., Shimo N., Takebe S., Watada H., Kaneto H., Matsuoka T. (2017). Recovered expression of Pdx1 improves β-cell failure in diabetic mice. Biochem. Biophys. Res. Commun..

[20] Zhu X., Oguh A., Gingerich M.A., Soleimanpour S.A., Stoffers D.A., Gannon M. (2021). Cell cycle regulation of the Pdx1 transcription factor in developing pancreas and insulin-producing beta cells. Diabetes.

[21] Zhang M., Yang C., Zhu M., Qian L., Luo Y., Cheng H., Geng R., Xu X., Qian C., Liu Y. (2021). Saturated fatty acids entrap PDX1 in stress granules and impede islet beta cell function. Diabetologia.

[22] van Genugten R.E., van Raalte D.H., Diamant M. (2012). Dipeptidyl peptidase-4 inhibitors and preservation of pancreatic islet-cell function: A critical appraisal of the evidence. Diabetes Obes. Metab..

[23] Hamamoto S., Kanda Y., Shimoda M., Tatsumi F., Kohara K., Tawaramoto K., Hashiramoto M., Kaku K. (2013). Vildagliptin preserves the mass and function of pancreatic beta cells via the developmental regulation and suppression of oxidative and endoplasmic reticulum stress in a mouse model of diabetes. Diabetes Obes. Metab..

[24] Hirukawa H., Kaneto H., Shimoda M., Kimura T., Okauchi S., Obata A., Kohara K., Hamamoto S., Tawaramoto K., Hashiramoto M. (2015). Combination of DPP-4 inhibitor and PPARγ agonist exerts protective effects on pancreatic β-cells in diabetic db/db mice through the augmentation of IRS-2 expression. Mol. Cell. Endocrinol..

[25] Wei R., Cui X., Feng J., Gu L., Lang S., Wei T., Yang J., Liu J., Le Y., Wang H. (2020). Dapagliflozin promotes beta cell regeneration by inducing pancreatic endocrine cell phenotype conversion in type 2 diabetic mice. Metabolism.

[26] Kusakabe T., Yokota S., Shimizu M., Inoue T., Tanaka M., Ohue-Kitano R., Muranaka K., Yamakage H., Wada H., Hasegawa K. (2020). Differential effects of sodium-glucose cotransporter 2 inhibitor and low-carbohydrate diet on body composition and metabolic profile in obese diabetic db/db mice. BMJ Open Diabetes Res. Care.

[27] Hogan M.F., Hackney D.J., Aplin A.C., Mundinger T.O., Larmore M.J., Castillo J.J., Esser N., Zraika S., Hull R.L. (2021). SGLT2-i improves markers of islet endothelial cell function in db/db diabetic mice. J. Endocrinol..

[28] Shu L., Matveyenko A.V., Kerr-Conte J., Cho J.H., McIntosh C.H., Maedler K. (2009). Decreased TCF7L2 protein levels in type 2 diabetes mellitus correlate with downregulation of GIP- and GLP-1 receptors and impaired beta-cell function. Hum. Mol. Genet..

[29] Xu G., Kaneto H., Laybutt D.R., Duvivier-Kali V., Trivedi N., Suzuma K., King G.L., Weir G.C., Bonner-Weir S. (2007). Downregulation of GLP-1 and GIP receptor expression by hyperglycemia: Possible contribution to the impaired incretin effects in diabetes. Diabetes.

[30] Kawashima S., Matsuoka T., Kaneto H., Tochino Y., Kato K., Yamamoto K., Yamamoto T., Matsuhisa M., Shimomura I. (2011). Effect of alogliptin, pioglitazone and glargine on pancreatic β-cells in diabetic db/db mice. Biochem. Biophys. Res. Commun..

[31] Liu Z., Habener J.F. (2008). Glucagon-like peptide-1 activation of TCF7L2-dependent Wnt signaling enhances pancreatic beta cell proliferation. J. Biol. Chem..

[32] Takamoto I., Kubota N., Nakaya K., Kumagai K., Hashimoto S., Kubota T., Inoue M., Kajiwara E., Katsuyama H., Obata A. (2014). TCF7L2 in mouse pancreatic beta cells plays a crucial role in glucose homeostasis by regulating beta cell mass. Diabetologia.

[33] Mitchell R.K., Mondragon A., Chen L., McGinty J.A., French P.M., Ferrer J., Thorens B., Hodson D.J., Rutter G.A., Xavier G.D. (2015). Selective disruption of Tcf7l2 in the pancreatic β cell impairs secretory function and lowers β cell mass. Hum. Mol. Genet..

[34] Jainandunsing S., Koole H.R., van Miert J.N.I., Rietveld T., Wattimena J.L.D., Sijbrands E.J.G., de Rooij F.W.M. (2018). Transcription factor 7-like 2 gene links increased in vivo insulin synthesis to type 2 diabetes. EBioMedicine.

[35] Nguyen-Tu M.S., da Silva Xavier G., Leclerc I., Rutter G.A. (2018). Transcription factor-7-like 2 (TCF7L2) gene acts downstream of the Lkb1/Stk11 kinase to control mTOR signaling, beta cell growth, and insulin secretion. J. Biol. Chem..

[36] Wu H.H., Li Y.L., Liu N.J., Yang Z., Tao X.M., Du Y.P., Wang X.C., Lu B., Zhang Z.Y., Hu R.M. (2019). TCF7L2 regulates pancreatic beta-cell function through PI3K/AKT signal pathway. Diabetol. Metab. Syndr..

[37] Zhang Z., Xu L., Xu X. (2021). The role of transcription factor 7-like 2 in metabolic disorders. Obes. Rev..

[38] Florez J.C., Jablonski K.A., Bayley N., Pollin T.I., de Bakker P.I., Shuldiner A.R., Knowler W.C., Nathan D.M., Altshuler D. (2006). TCF7L2 polymorphisms and progression to diabetes in the Diabetes Prevention Program. N. Engl. J. Med..

[39] Horikoshi M., Hara K., Ito C., Nagai R., Froguel P., Kadowaki T. (2007). A genetic variation of the transcription factor 7-like 2 gene is associated with risk of type 2 diabetes in the Japanese population. Diabetologia.

[40] Lyssenko V., Lupi R., Marchetti P., Lyssenko V., Lupi R., Marchetti P., del Guerra S., Orho-Melander M., Almgren P., Sjögren M. (2007). Mechanisms by which common variants in the TCF7L2 gene increase risk of type 2 diabetes. J. Clin. Investig..

[41] Boj S.F., van Es J.H., Huch M., Li V.S., Jose A., Hatzis P., Mokry M., Haegebarth A., van den Born M., Chambon P. (2012). Diabetes risk gene and Wnt effector Tcf7l2/TCF4 controls hepatic response to perinatal and adult metabolic demand. Cell.

[42] Villareal D.T., Robertson H., Bell G.I., Patterson B.W., Tran H., Wice B., Polonsky K.S. (2010). TCF7L2 variant rs7903146 affects the risk of type 2 diabetes by modulating incretin action. Diabetes.

[43] Seino S., Takahashi H., Fujimoto W., Shibasaki T. (2009). Roles of cAMP signalling in insulin granule exocytosis. Diabetes Obes. Metab..

[44] Yu Z., Jin T. (2010). New insights into the role of cAMP in the production and function of the incretin hormone glucagon-like peptide-1 (GLP-1). Cell. Signal..

[45] Tengholm A., Gylfe E. (2017). cAMP signalling in insulin and glucagon secretion. Diabetes Obes. Metab..

[46] Alhosaini K., Azhar A., Alonazi A., Al-Zoghaibi F. (2021). GPCRs: The most promiscuous druggable receptor of the mankind. Saudi Pharm. J..

[47] Shigeto M., Ramracheya R., Tarasov A.I., Cha C.Y., Chibalina M.V., Hastoy B., Philippaert K., Reinbothe T., Rorsman N., Salehi A. (2015). GLP-1 stimulates insulin secretion by PKC-dependent TRPM4 and TRPM5 activation. J. Clin. Investig..

[48] Kaku K. (2020). New concept of the glucagon-like peptide-1 signaling pathway on pancreatic insulin secretion. J. Diabetes Investig..

[49] Shimoda M., Kanda Y., Hamamoto S., Tawaramoto K., Hashiramoto M., Matsuki M., Kaku K. (2011). The human glucagon-like peptide-1 analogue liraglutide preserves pancreatic beta cells via regulation of cell kinetics and suppression of oxidative and endoplasmic reticulum stress in a mouse model of diabetes. Diabetologia.

[50] Cernea S., Raz I. (2011). Therapy in the early stage: Incretins. Diabetes Care.

[51] Kimura T., Kaneto H., Shimoda M., Hirukawa H., Hamamoto S., Tawaramoto K., Hashiramoto M., Kaku K. (2015). Protective effects of pioglitazone and/or liraglutide on pancreatic β-cells: Comparison of their effects between in an early and advanced stage of diabetes. Mol. Cell. Endocrinol..

[52] Tamura K., Minami K., Kudo M., Iemoto K., Takahashi H., Seino S. (2015). Liraglutide improves pancreatic beta cell mass and function in Alloxan-induced diabetic mice. PLoS ONE.

[53] Zheng J., Chen T., Zhu Y., Li H.-Q., Deng X.-L., Wang Q.-H., Zhang J.-Y., Chen L.-L. (2015). Liraglutide prevents fast weight gain and β-cell dysfunction in male catch-up growth rats. Exp. Biol. Med..

[54] Kapodistria K., Tsilibary E.-P., Kotsopoulou E., Moustardas P., Kitsiou P. (2018). Liraglutide, a human glucagon-like peptide-1 analogue, stimulates AKT-dependent survival signalling and inhibits pancreatic β-cell apoptosis. J. Cell. Mol. Med..

[55] Gao M., Deng X.-L., Liu Z.-H., Song H.-J., Zheng J., Cui Z.-H., Xiao K.-L., Chen L.-L., Li H.-Q. (2018). Liraglutide protects β-cell function by reversing histone modification of Pdx-1 proximal promoter in catch-up growth male rats. J. Diabetes Complicat..

[56] Fushimi Y., Obata A., Sanada J., Nogami Y., Ikeda T., Yamasaki Y., Obata Y., Shimoda M., Nakanishi S., Mune T. (2021). Combination of dipeptidyl peptidase 4 (DPP-4) inhibitor and sodium glucose cotransporter 2 (SGLT2) inhibitor substantially protects pancreatic β-cells especially in early phase of diabetes rather than advanced phase. Sci. Rep..

[57] Kimura T., Obata A., Shimoda M., Hirukawa H., Kanda-Kimura Y., Nogami Y., Kohara K., Nakanishi S., Mune T., Kaku K. (2018). Durability of protective effect of dulaglutide on pancreatic β-cells in diabetic mice: GLP-1 receptor expression is not reduced at all even after long-term exposure to dulaglutide. Diabetes Metab..

[58] Heber D., Dodson R., Stoskopf C., Peterson M., Swerdloff R.S. (1982). Pituitary desensitization and the regulation of pituitary gonadotropin-releasing hormone (GnRH) receptors following chronic administration of a superactive GnRH analog and testosterone. Life Sci..

[59] He L., Fong J., von Zastrow M., Whistler J.L. (2002). Regulation of opioid receptor trafficking and morphine tolerance by receptor oligomerization. Cell.

[60] Conn P.M., Crowley W.F. (1991). Gonadotropin-releasing hormone and its analogues. N. Engl. J. Med..

[61] Abu Hashim H. (2012). Gonadotrophin-releasing hormone analogues and endometriosis: Current strategies and new insights. Gynecol. Endocrinol..

[62] Arakawa M., Mita T., Azuma K., Ebato C., Goto H., Nomiyama T., Fujitani Y., Hirose T., Kawamori R., Watada H. (2010). Inhibition of monocyte adhesion to endothelial cells and attenuation of atherosclerotic lesion by a glucagon-like peptide-1 receptor agonist, exendin-4. Diabetes.

[63] Goto H., Nomiyama T., Mita T., Yasunari E., Azuma K., Komiya K., Arakawa M., Jin W.L., Kanazawa A., Kawamori R. (2011). Exendin-4, a glucagon-like peptide-1 receptor agonist, reduces intimal thickening after vascular injury. Biochem. Biophys. Res. Commun..

[64] Wang D., Jiang L., Feng B., He N., Zhang Y., Ye H. (2020). Protective effects of glucagon-likepeptide-1 on cardiac remodeling by inhibiting oxidative stress through mammalian target of rapamycin complex 1/p70 ribosomal protein S6kinase pathway in diabetes mellitus. J. Diabetes Investig..

[65] Helmstadter J., Frenis K., Filippou K., Grill A., Dib M., Kalinovic S., Pawelke F., Kus K., Kröller-Schon S., Oelze M. (2020). Endothelial GLP-1 (glucagon-like peptide-1) receptor mediates cardiovascular protection by liraglutide in mice with experimental arterial hypertension. Arterioscler. Thromb. Vasc. Biol..

[66] Kimura T., Obata A., Shimoda M., Okauchi S., Hirukawa H., Kohara K., Kinoshita T., Nogami Y., Nakanishi S., Mune T. (2017). Decreased GLP-1 receptor expression in endothelial and smooth muscle cells in diabetic *db/db* mice: TCF7L2 is a possible regulator of vascular GLP-1 receptor. Diabetes Vasc. Dis. Res..

[67] Kimura T., Obata A., Shimoda M., Shimizu I., da Silva Xavier G., Okauchi S., Hirukawa H., Kohara K., Mune T., Moriuchi S. (2018). Down-regulation of vascular GLP-1 receptor expression in human subjects with obesity. Sci. Rep..

[68] Sanada J., Obata A., Obata Y., Fushimi Y., Shimoda M., Kohara K., Nakanishi S., Mune T., Kaku K., Kaneto H. (2021). Dulaglutide exerts more beneficial anti-atherosclerotic effects in an early phase of diabetes rather than in a late phase in ApoE knockout mice with diabetes. Sci. Rep..

[69] Marso S.P., Daniels G.H., Brown-Frandsen K., Kristensen P., Mann J.F., Nauck M.A., Nissen S.E., Pocock S., Poulter N.R., Ravn L.S. (2016). Liraglutide and cardiovascular outcomes in type 2 diabetes. N. Engl. J. Med..

[70] Verma S., Poulter N.R., Bhatt D.L., Bain S.C., Buse J.B., Leiter L.A., Nauck M.A., Pratley R.E., Zinman B., Ørsted D.D. (2018). Effects of liraglutide on cardiovascular outcomes in patients with type 2 diabetes mellitus with or without history of myocardial infarction or stroke. Circulation.

[71] Gerstein H.C., Colhoun H.M., Dagenais G.R., Diaz R., Lakshmanan M., Pais P., Probstfield J., Riesmeyer J.S., Riddle M.C., Rydén L. (2017). Dulaglutide and cardiovascular outcomes in type 2 diabetes (REWIND): A double-blind, randomised placebo-controlled trial. Lancet.

[72] Kristensen S.L., Rørth R., Jhund P.S., Docherty K.F., Sattar N., Preiss D., Køber L., Petrie M.C., McMurray J.J.V. (2019). Cardiovascular, mortality, and kidney outcomes with GLP-1 receptor agonists in patients with type 2 diabetes: A systematic review and meta-analysis of cardiovascular outcome trials. Lancet Diabetes Endocrinol..

[73] Marso S.P., Bain S.C., Consoli A., Eliaschewitz F.G., Jódar E., Leiter L.A., Lingvay I., Rosenstock J., Seufert J., Warren M.L. (2016). Semaglutide and cardiovascular outcomes in patients with type 2 diabetes. N. Engl. J. Med..

[74] Kaul S. (2017). Mitigating cardiovascular risk in type 2 diabetes with antidiabetes drugs: A review of principal cardiovascular outcome results of EMPA-REG OUTCOME, LEADER, and SUSTAIN-6 trials. Diabetes Care.

